# Time delays and capability of elderly to activate speaker function for continuous telephone CPR

**DOI:** 10.1186/1757-7241-21-40

**Published:** 2013-05-15

**Authors:** Tonje S Birkenes, Helge Myklebust, Jo Kramer-Johansen

**Affiliations:** 1 Institute for Experimental Medical Research, Oslo University Hospital and University of Oslo, PO Box 4956 Nydalen, Oslo, N-0426, Norway; 2 Laerdal Medical AS, Tanke Svilandsgate 30, Stavanger, N-4002, Norway

## Abstract

**Background:**

Telephone-CPR (T-CPR) can increase rate of bystander CPR as well as CPR quality. Instructions for T-CPR were developed when most callers used a land line. Telephones today are often wireless and can be brought to the patient. They often have speaker function which further allows the rescuer to receive instructions while performing CPR.

We wanted to measure adult lay people’s ability to activate the speaker function on their own mobile phone.

**Methods:**

Elderly lay people, previously trained in CPR, were contacted by telephone. Participants with speaker function experience were asked to activate this without further instructions, while participants with no experience were given instructions on how to activate it. Participants were divided in three groups; Group 1: Can activate the speaker function without instruction, Group 2: Can activate the speaker function with instruction, and Group 3: Unable to activate the speaker function. Time to activation for group 1 and 2 was compared using Mann-Whitney U-test.

**Results:**

Seventy-two elderly lay people, mean age 68 ± 6 years participated in the study. Thirty-five (35)% of the participants were able to activate the speaker function without instructions, 29% with instructions and 36% were unable to activate the speaker function. The median time to activate the speaker function was 8s and 93s, with and without instructions, respectively (p < 0.01).

**Conclusion:**

One-third of the elderly could activate speaker function quickly, and two-third either used a long time or could not activate the function.

## Background

Most out-of-hospital cardiac arrests (OHCAs) occur in adults above 50 years old [1,2]. In 90% of cases, the victim and the bystander know each other [3], 70% occurs at home [3,4], and the rescuer is often alone with the victim [3].

Instructions by emergency service dispatchers to start CPR and to improve quality of CPR via telephone (T-CPR) were first initiated in King County in 1983 [5], and resulted in 50% increase in bystander CPR [6]. This increased rate of bystander CPR has later been confirmed elsewhere [2,7,8]. T-CPR was strongly recommended by the International Liaison Committee on Resuscitation in 2010 [9], and reinforced by the American Heart Association in 2012 [10]. T-CPR might also improve CPR quality [11], and good quality bystander CPR is associated with improved survival [12-14].

Initial dispatcher instructions were developed when most callers used a land line as illustrated in the Norwegian guidelines 2005 [15-17]. Using a land line results in instructions and CPR occurring sequentially, as the rescuer alternates between patient and telephone [18]. Activation of the speaker function of a wireless telephone may allow the rescuer to receive instructions and encouragement from the dispatcher simultaneously with performing CPR [19-21], but to our knowledge, no studies have tested lay people’s ability to activate the speaker function on their own mobile phone.

Today, most people in the developed world and a rapidly increasing portion in developing countries have a mobile phone. The first hand-held portable cell phone was presented in 1973, and the pocket version of mobile phones was developed and introduced in the late 80’s. As more and more functions were added to mobile phones, Nokia introduced their speaker function in the 90’s and today this is standard on most mobile phones.

In a pilot study, we found that 30 of 35 lay people between 16 and 60 years old were able to activate the speaker function within 5 s without instructions [22]. We wanted to measure ability and time delays to activate speaker function on personal mobile phones in an elderly population, presumably more likely to witness a cardiac arrest.

## Method

### Study design

Observational study of older adults with their own mobile phones.

### Recruitment of participants and test situation

One hundred and thirty-one (131) volunteers aged 50 years and older who had attended CPR training in 2008 and signed up or participated in a CPR research study in 2009 [21,23], accepted to be contacted to participate in further research. This group received a written note by mail, informing them we would call to ask for help on a research question. Seventy-four (74) out of 131 answered the phone and were included in the study. The participants were reminded that participation was voluntarily and that all data would be treated anonymously.

**Figure 1:**
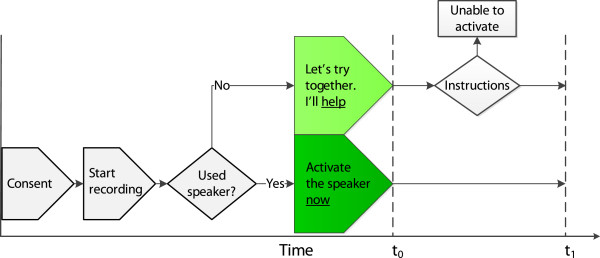
**Timeline.** t_0_ = time when participant was asked to activate the speaker or start of instructions for those with no previous speaker function experience. t_1_ = time when speaker function confirmed activated.

The participants were asked to participate in a study with the topic “use of mobile phone”. If the participant accepted, she or he was requested to hang up and then return the call to the researcher. Verbal consent was then given, and the conversation was recorded using Audacity ver.2.0 (http://audacity.sourceforge.net). The researcher asked *“Have you ever used the speaker function on your mobile phone?”* If “yes”, the researcher continued *“Would you please activate the speaker function now (t*_*0*_*) and tell me when it’s done?”* If *“no”*, the researcher gave the following brief instructions: *“Let’s try this together, I’ll help (t*_*0*_*) you. Look at the screen. Do you see a symbol for speaker function or the word speaker? If so, press on the symbol or the button that is closest” *(Figure 1). If the speaker function was not readily available by one press of a button, the participant was instructed to look under menu or other shortcuts available on the screen.

### Data collection and statistical analysis

The participants were grouped into

1. Can activate speaker function without instructions

2. Can activate speaker function with instructions

3. Unable to activate speaker function

Time to activated speaker function in groups 1 and 2 was defined as t_1_ – t_0_, where t_0_ was the time when we asked the participant to activate the speaker function and t_1_ was when the activated speaker function was confirmed verbally, both determined from audio recordings (see Figure 1 for timeline and overview of the test setting). Data on demography, education level and mobile phone brand was collected.

Difference in median time to activated speaker function was analyzed using Mann-Whitney U-test. Results are presented as mean (standard deviation) or median [25% percentile, 75% percentile] as appropriate. We also tested if there was any association between more advanced age and ability to activate speaker phone by a Chi-square test of proportions above and below the median age of 69. SPSS ver. 20 (SPSS Inc., Chicago, IL) was used for statistical analysis.

## Results

Seventy-two (72) of 74 people contacted, completed the study with mean age 68 ± 6 years (Table 1). One participant did not return the call to the researcher (see Method) and one subject withdrew during the test without specifying a reason. The study was conducted in Stavanger, Norway in March-April 2012.

**Table 1:** Demography and background

**Age**	**54 - 68**	**> = 69**
Number of participants	41	31
Female	23	19
Completed education*		
Elementary/high school	6	9
Occupational school	16	12
Lower university grade	10	11
Higher university grade	2	5
Mobile phone*		
Nokia	18	26
iPhone	7	2
Doro**	3	3
Other	6	6

* Missing data for one participant.

** Designed for seniors.

**Figure 2:**
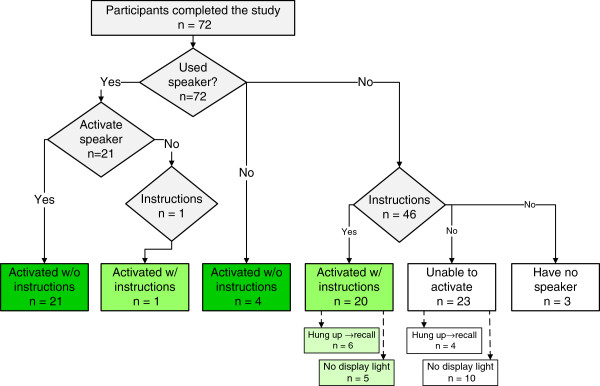
**Flow diagram of participants.** Flow diagram of participants illustrating the distribution of the results. Without and with is abbreviated to w/o and w/, respectively.

**Figure 3:**
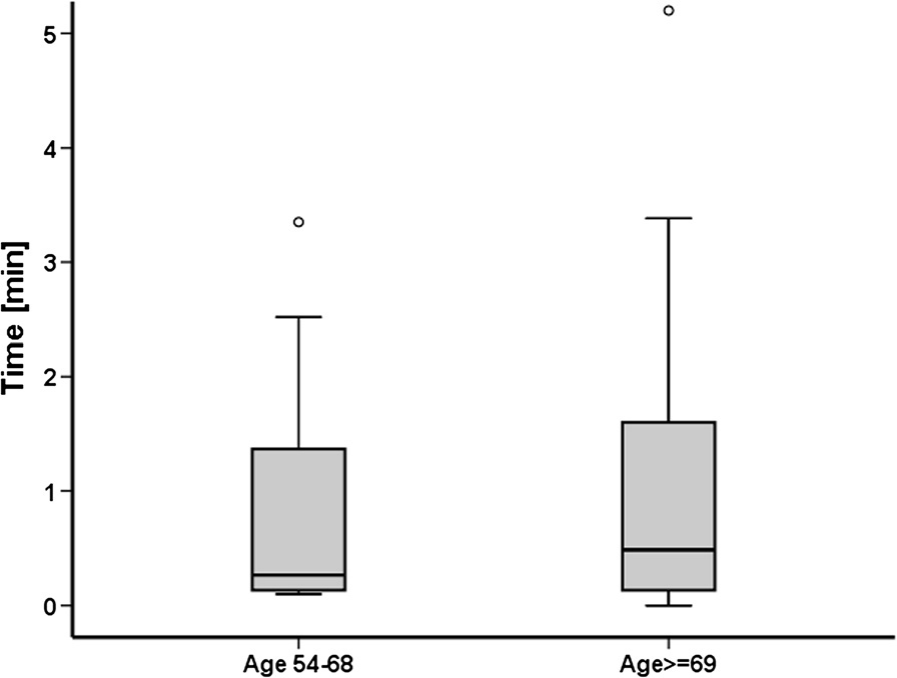
**Box plot.** Box plot of median time to activate speaker function sorted in age groups 50-69 and above 69.

Twenty-five (25) of 72 (35%) participants managed to activate the speaker function without instructions and 21 (29%) with instructions, with median times 8 s [7,16] and 93 s [39, 127] (p < 0.01), respectively. Twenty-six (36%) were unable to activate the speaker despite given instructions, all of whom had no previous experience activating the speaker function (Figure 2, Table 2). Some participants claimed the mobile phone did not have speaker function (Figure 2). There was no significant difference between the age groups above and below the median age of 69, in the capability to activate the speaker function without instruction (Chi-square test, p = 0.16, Table 2), nor in time to activate the speaker the speaker function (Mann-Whitney, p = 0.61, see Figure 3 for box plot).

**Table 2:** Results for ability to activate speaker function vs. age group

	**Age 54 - 68**	**Age ≥69**	**p-value for difference between age groups**
	**n = 35**	**n = 37**	
Activated without instructions	15	10	0.16
Activated with instructions	8	13	0.25
Unable to activate	12	14	0.75

Six (6) of 21 participants who needed instructions (Group 2) and four in the group that never managed to activate speaker function (Group 3), unintentionally hung up and had to be called back to repeat the test (Figure 2). A total of 15 participants that were offered instructions reported that the display had turned black at the time when they should activate the speaker function.

## Discussion

Survival after cardiac arrest decreases rapidly with every minute before CPR is initiated [4,24], and all delays should be minimized. Single-rescuer situations are most common [3], and median 93 s to activate the speaker function for the third of participants who were able to do so with instructions, is a significant delay. It is even more problematic that another third were unable to activate the speaker function. On the other hand, it is encouraging that those elderly lay persons with previous speaker function experience were able to activate it within median 8 seconds. This is a strong indication that speaker phone activation is feasible and the topic should be included as an integral part of all lay person CPR training.

With speaker function activated, the rescuer can assess the patient, identify cardiac arrest, and initiate CPR without interrupting continuous contact with the dispatcher. This enables integrated instructions and rescuer actions instead of instructions and actions occurring sequentially. With continuous CPR instructions, CPR quality can improve, which we know improves survival [12-14].

We have previously observed that 30 out of 31 adult lay people managed to perform CPR and communicate with the dispatcher at the same time, in a realistic simulated cardiac arrest scenario. CPR quality improved or did not deteriorate for 10 minutes of single rescuer resuscitation [11]. Real-time encouragement from a dispatcher provides rescuers with mental and emotional support, [25] and it allows dispatcher and rescuer to work as a team, where the dispatcher is the team leader. By offering continuous instructions, the dispatcher relieves the feeling of being alone in a highly stressful situation [11,21].

### Time to first compression

The time to first compression with dispatcher instructed CPR has been discouragingly long in previous studies. In a recent clinical study of Medical Priority Dispatch System (MPDS) protocols the mean delay before first compression was four minutes in 519 cardiac arrest calls even after instructions regarding pulse check and mouth-to-mouth ventilation had been removed. [26] These results were no better than the four-minute delays reported by the Seattle group in 2003 when their instructions included ventilations [3]. Hallstrom did not report time to first chest compression, but found that delivering CPR instructions took 165 seconds if including airways and ventilation vs. 79 seconds for chest compressions alone [27].

It is unknown if time spent on instructions to activate the speaker will delay first compression. The present results indicate that a delay is likely in elderly callers not familiar with the activation technique. On the other hand, once continuous speaker phone communication is established, it is possible that dispatcher and rescuer collaborates better and are able to initiate CPR faster. This should be further investigated. The balance between potential delays to first chest compression versus the benefit of improving CPR quality is also unknown.

### Ability to activate the speaker function

Hauff reported that 47% of actual bystanders were in age group 36-65, and 41% in age group >65 [3]. Our initial expectations were that the majority of those who activated the speaker function without instructions would be in the age group 50 – 68, but the older group was equally able to activate the speaker function, provided they had done it before (Table 2).

In our study, only one or two operations were necessary to activate the speaker function, and the procedure was not unified between the different phone models. A future option could be that the dispatcher can remotely put the callers’ phone on speaker. This would ensure speaker activation, eliminate time expenditure and mitigate the risk that callers unintentionally hang up, which happened in 14% of our cases. It would also help those who are unable to read the display due to small letters (or symbols), impaired sight, or bright light conditions. A concern associated with speaker phone option is background noise and impaired communication. As single-rescuer situations in a home are the most common for sudden out-of-hospital cardiac arrests [3], the background noise will probably not be that disturbing. In the present test, all participants were able to communicate with the researcher.

### Today’s situation

Current dispatcher protocols do not include speaker function activation [17], but it is unknown what proportion of callers or dispatchers who initiate activation of speaker function on their own initiative.

Although introduced already in 1984 [5], current T-CPR coverage is limited in many places [10]. Prerecorded instructions and just-in-time education from a smart phone might be an alternative in the absence of T-CPR [28-30], but to our knowledge only T-CPR has shown to increase bystander CPR rates and outcome [2].

### Recommendations

CPR training materials and dispatcher protocols should actively consider how best to incorporate the speaker mode into telephone CPR instruction, allowing rescuers to receive instructions and help from the dispatcher and provide CPR at the same time.

Campaigns may be used to highlight the potential of this functionality and how it can be activated. This may encourage people to become familiar with their own speaker function and become confident using it when calling medical emergency response.

The phone models used by our participants had different procedures to activate the speaker function. Manufacturers should agree to simple and unified steps to make it easier for dispatchers to instruct the rescuer to activate the speaker and for people to become confident using it.

### Limitations

Our study was performed in a controlled environment, without induced stress. As real situations can be stressful, pressing the wrong button and disconnecting with the dispatcher is probably more likely to happen.

Our study population was previously CPR trained, and may perform better in general than the average caller, although activation of speaker function was not part of their previous training or testing.

The mobile phone needs to be readily available to be used in an emergency situation. We did not ask our participants where they normally kept their mobile phone, but this should be further investigated.

In our study there was no significant difference between time to activation of speaker function associated with increased age, gender nor mobile phone brand. However, we cannot exclude the possibility for a type II error and neither that a larger sample might allow for multivariate analysis of other factors that might predict the ability to activate speaker function.

We only tested the ability to activate the speaker function, and did not continue with dispatcher-assisted telephone-CPR. This should be further investigated to learn more about how dispatcher-rescuer teamwork can improve the quality of bystander CPR.

## Conclusion

Elderly lay people with mobile speaker function experience activated the function quickly. Elderly without such experience either used long time or could not activate speaker function.

## Competing interests

Birkenes receive research scholarships provided by the Norwegian Research Council. Birkenes and Myklebust are employees at Laerdal Medical. Kramer-Johansen receives financial research support from Laerdal Medical. The study was sponsored by Laerdal Medical, Stavanger, Norway.

## Authors’ contributions

All authors participated in the study design. TSB and HM collected the data; TSB performed the statistical analysis and drafted the manuscript. All authors have critical reviewed the manuscript, and the study was supervised by JKJ. All authors read and approved the final manuscript.
